# Clinical application of intramedullary nail fixation assisted by locking plates in segmental tibia fractures: A retrospective study

**DOI:** 10.1097/MD.0000000000040855

**Published:** 2024-12-13

**Authors:** Wangsheng Wu, Huajuan Wang, Qunyang Zheng, Yi Mao, Bingsheng Liu

**Affiliations:** a Quzhou People’s Hospital, The Quzhou Affiliated Hospital of Wenzhou Medical University, Quzhou, Zhejiang, China.

**Keywords:** intramedullary nail, locking plate, open reduction, tibia fractures

## Abstract

It is unclear whether small plates are needed for augment fixation to provide a more stable mechanical environment in segmental fractures of the tibia treated with intramedullary nail. The purpose of this study was to investigate the efficacy of intramedullary nailing combined with locking plates in the treatment of segmental fractures of the tibia. This study included 41 patients with segmental tibia fractures between January 1, 2018 and January 1, 2023. Eighteen patients were treated with an intramedullary nail assisted by a locking plate (combination group), and 23 patients were treated with an intramedullary nail without a plate (nail group). The perioperative parameters of all patients were recorded, and clinical efficacy was evaluated during follow-up. The operation time was shorter but the incision was longer in the combination group (*P* < .05). The numbers of fluoroscopy procedures and the time to union were shorter in the combination group (*P* < .05). The rate of malunion in the nail group (4, 17.4%) was significantly greater than that in the combination group (0, 0%). At the last follow-up, no statistically significant differences in walking ability were detected. Our results suggest that a locking plate combined with an intramedullary nail for segmental tibial fractures may require a longer surgical incision, but it has significant advantages such as a shorter operative time and time to union, a shorter fluoroscopy time, and a lower rate of malunion and nonunion. Intramedullary nail fixation assisted by a locking plate is an effective method for treating segmental fractures of the tibia.

## 1. Introduction

Tibial shaft fracture is the most common fracture of long tubular bones.^[1,2]^ The tibia and fibula take the force from the femur/patella and transmit it to the ankle joint to achieve a normal walking gait. Since the knee and ankle joints are hinge joints, the rotation and lateral angulation deformities after tibial fracture cannot be adjusted. Intramedullary nail (IMN) remains the preferred procedure for the treatment of unstable and displaced tibial shaft fractures because it can act as a load-sharing device, providing fracture biomechanical stability and allowing early patients mobilization.^[3]^ However, the incidence of malrotation after treatment with IMN in tibial fractures has been reported to be as high as 30%.^[4–6]^ The anterior medial tibia is covered only by skin, and its blood supply is worse than that of other long bones; thus, the incidence of nonunion is higher when tibia fractures are improperly treated, especially segmental tibia fractures (STF).

Despite the increasing understanding of the anatomy and biomechanics of the tibia, the treatment of STF remains tricky for orthopedists. The goal of treatment for STF is to achieve anatomic reduction and rigid fixation. Accurate reduction and early functional exercise are key to restoring the physiological function of the limbs. However, owing to the long intermediate fragment, it is difficult to completely correct the deformation via closed reduction, and it is difficult to achieve stable fixation for bone healing with the IMN alone.

The IMN is representative of central fixation. The plate is representative of eccentric fixation, and the biomechanics are different. However, in recent years, an increasing number of IMN combined with plates have been used to treat some complex periarticular fractures and postoperative nonunions, and satisfactory results have been obtained. However, whether this combination can be used in STF is unknown. This study aimed to evaluate the effectiveness and safety of combining IMN with plate fixation for the treatment of STF by comparing the clinical outcomes with those of IMN alone.

## 2. Materials and methods

**Figure 1.**
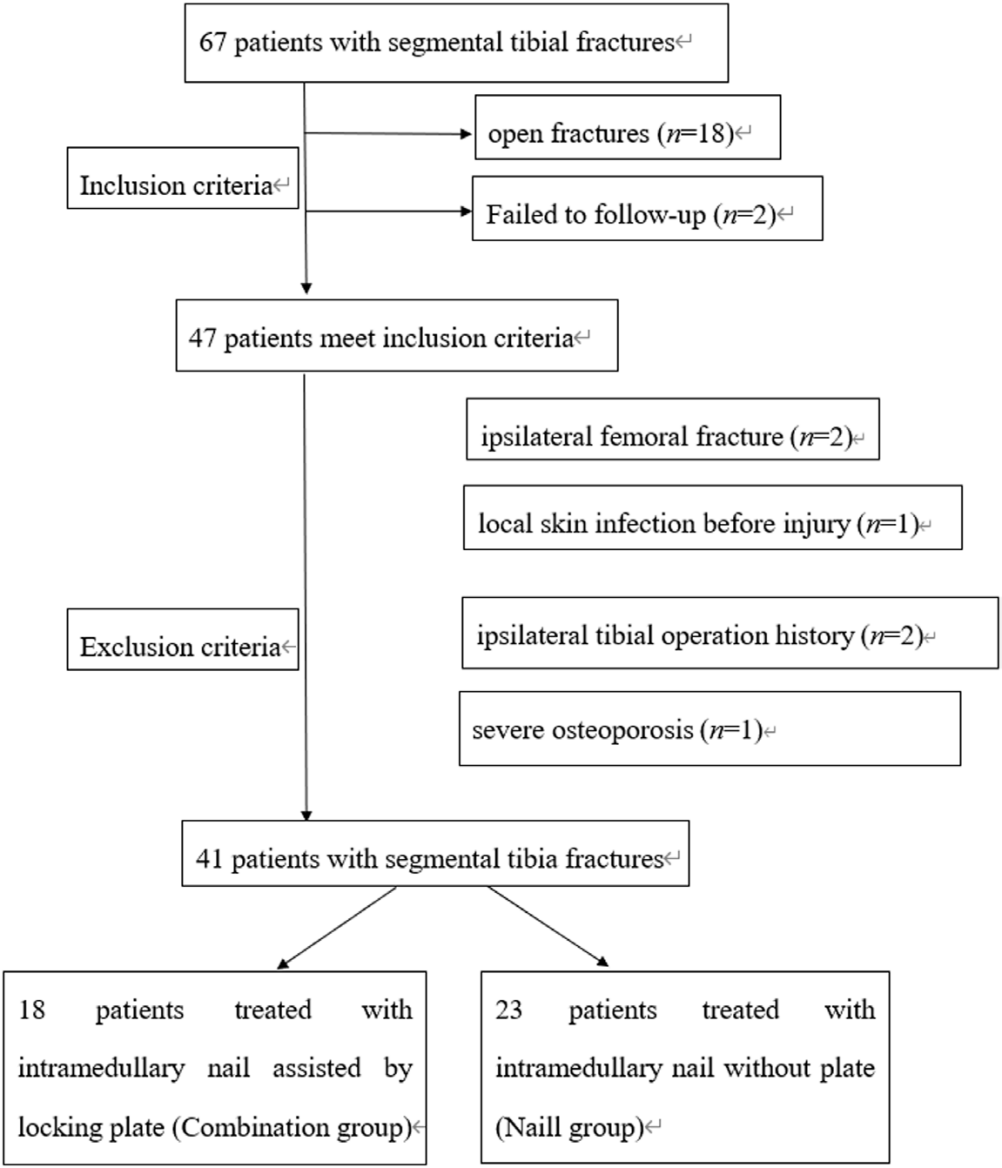
Flowchart of the inclusion and exclusion criteria.

This study was approved by the Medical Ethics Review Committee of the Quzhou Affiliated Hospital of Wenzhou Medical University, Quzhou People’s Hospital and informed consent was obtained from all patients. This retrospective study was conducted from January 2024 through February 2024. The study was conducted on all adult (>18 years of age) patients with STF from January 1, 2018 to January 1, 2023. The inclusion criteria were as follows: fresh STF; closed injuries; and at least 1 year of follow-up. The exclusion criteria were as follows: local skin infection before injury; ipsilateral femoral fracture; pathological fractures; ipsilateral tibial operation history; and severe osteoporosis (T score ≤ −2.5 SD). A total of 41 patients were eligible for this study, including 18 patients treated with IMN combined with plates (combination group) and 23 patients treated with IMN only (nail group; Fig. 1).

### 2.1. Perioperative procedures

#### 2.1.1. Combination group

The patients laid in a supine position on a radiolucent table. The incision was centered on the fracture site and located 1 cm lateral to the tibial crest. The anterior tibial muscle was pulled laterally to expose the fracture and then reduced the fracture. The procedure was performed carefully to minimize blood loss. After reduction, the fracture was temporarily fixed with Kirschner wire (2.0 mm), and then a locking plate (2.4 mm, Wego, China) was placed on the anterolateral surface. Two or 3 locking screws were placed at each side of the fracture. The screw length was between 8 and 10 mm, and the length of the screw could not exceed the thickness of the cortical bone because long screws may affect subsequent IMN placement. However, the screws could not be too short or else the procedure would fail. After the plate was placed, the reduction quality was determined via fluoroscopy. Next, traditional antegrade tibial nailing was performed. Violent manipulation was strictly prohibited during reaming and IMN placement to prevent redisplacement of the fracture. The incision was sutured after the drainage tube was filled.

#### 2.1.2. Nail group

The patients laid supine on a radiolucent table. In most cases, owing to the difficulty in correcting the rotation and angulation of the long intermediate fragment, it is almost impossible to achieve satisfactory reduction with closed reduction. Therefore, we used assistive devices or assistive technologies such as clamps, Schanz pins, “joystick” techniques, and poller screws to help reduce the risk. After fracture reduction, traditional antegrade tibial nailing was performed slowly and carefully to prevent fracture displacement.

### 2.2. Rehabilitation protocols

Prophylactic antibiotics were given 30 minutes before surgery (cefuroxime, 1.5 g), and intravenous antibiotics were given within 24 hours after surgery (cefuroxime, 1.5 g, 3 times). The drainage tube was usually removed at 48 hours after surgery, and then passive exercises were started on adjacent joints, including the knee and ankle joints. In the combination group, toe-touch weight bearing exercise were carried out after the patient’s condition was stable, approximately 1 week after surgery. In the nail group, toe-touch weight bearing exercises were carried out approximately 4 weeks after surgery, and all patients progressed to full weight bearing exercises thereafter.

### 2.3. Radiological evaluation

Malreduction or malunion was defined as angulation >5 degrees in the coronal plane or >10 degrees in the sagittal plane according to radiologic outcomes.^[7,8]^ Fracture union was defined by symptoms and images, including painless walking with full weight-bearing and cortical healing of at least 3 cortices on the AP view and lateral view on the radiographs. Fracture union was determined by 2 orthopedic surgeons. In cases of disagreement between the 2 orthopedic surgeons, the decision was made by a third senior orthopedic surgeon.

### 2.4. Patient follow-up

The sex, age, and other characteristics of the patients were evaluated. Perioperative parameters such as the incision length, operation time, amount of intraoperative blood loss and number of fluoroscopies were recorded. X-rays were taken after the drainage tube was removed, and patients were usually discharged approximately 1 week after surgery. In the first half year after discharge, patients visited the hospital every 4 weeks. The patients then returned to the hospital every 6 months. X-ray examination and instructed functional exercise were carried out at each follow-up. Walking ability was assessed at the 1-year follow-up. If the patients required revision surgery, an evaluation was performed 1 year after revision surgery. Walking ability was graded from 0 to 9.^[9]^ All postoperative complications were recorded.

### 2.5. Statistical analysis

IBM SPSS Statistics 20 (IBM Corp., Armonk) was used for analysis. Continuous variables were expressed as the mean ± standard deviation, and categorical variables were expressed as absolute values and percentages. Associations between categorical variables were tested by χ^2^ or Fisher exact tests. Relationships between continuous variables were tested by independent samples *t* test, and non-normal distribution was tested by the Mann–Whitney *U* test. Statistical significance was defined as *P* < .05.

## 3. Results

**Figure 2.**
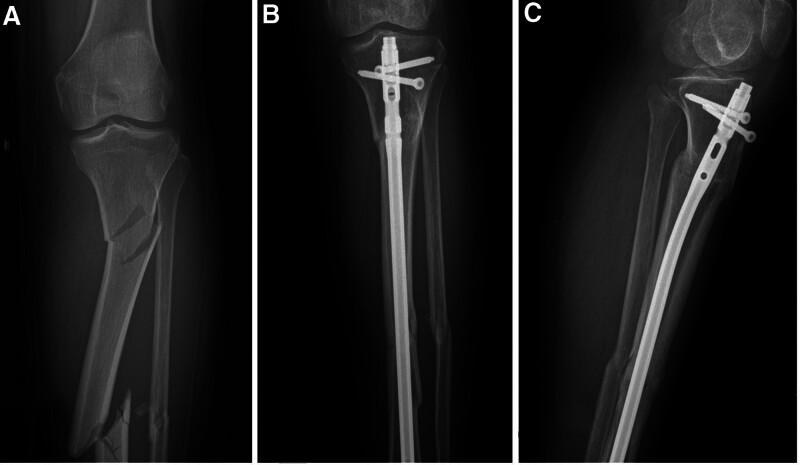
(A) A 65-year-old male patient involved in a traffic accident with STF. (B) Six months after internal fixation with the IMN, the anteroposterior image revealed malunion. (C) Six months after internal fixation with the IMN, the lateral image revealed malunion. IMN = intramedullary nail, STF = segmental tibia fractures.

**Figure 3.**
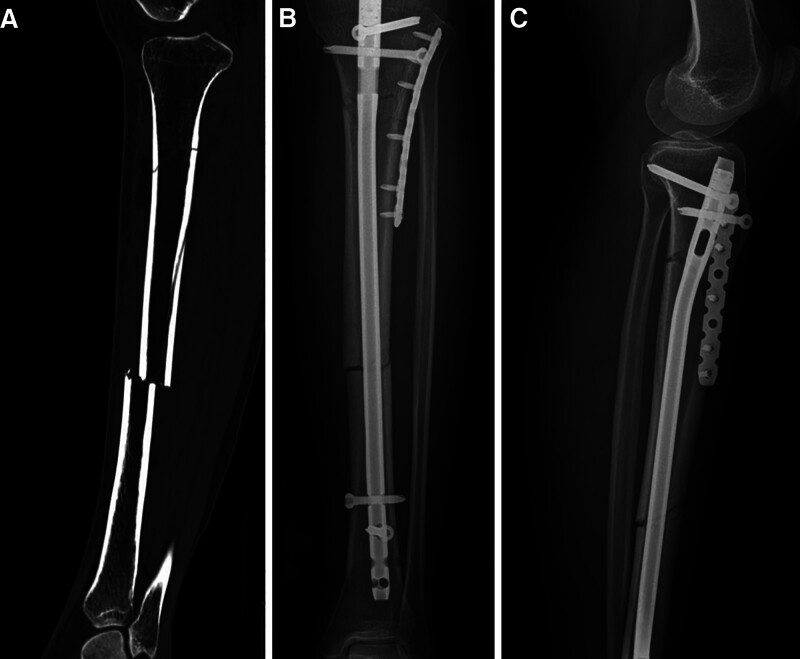
(A) A 43-year-old female patient sustained a left STF. (B) After internal fixation with an IMN assisted by a monocortical locking plate, the anteroposterior image showed anatomic reduction. (C) The lateral image also showed anatomic reduction. IMN = intramedullary nail, STF = segmental tibia fractures.

**Figure 4.**
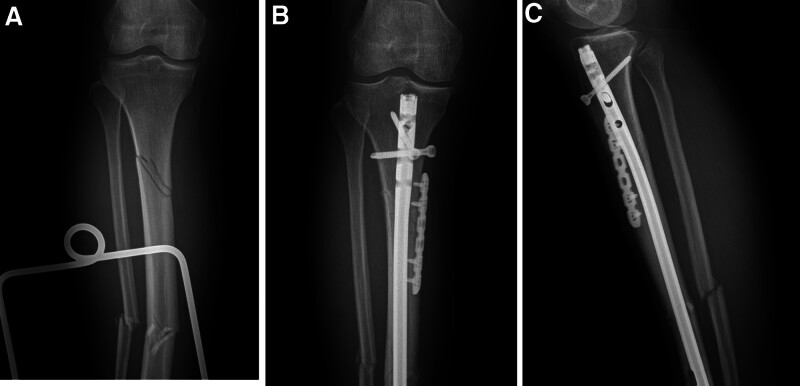
(A) A 59-year-old female patient involved in a traffic accident with STF. (B) Two months after internal fixation with an IMN assisted by a monocortical locking plate, the anteroposterior image showed union and good reduction. (C) Two months after internal fixation with an IMN assisted by a monocortical locking plate, the lateral image showed union and good reduction. IMN = intramedullary nail, STF = segmental tibia fractures.

**Figure 5.**
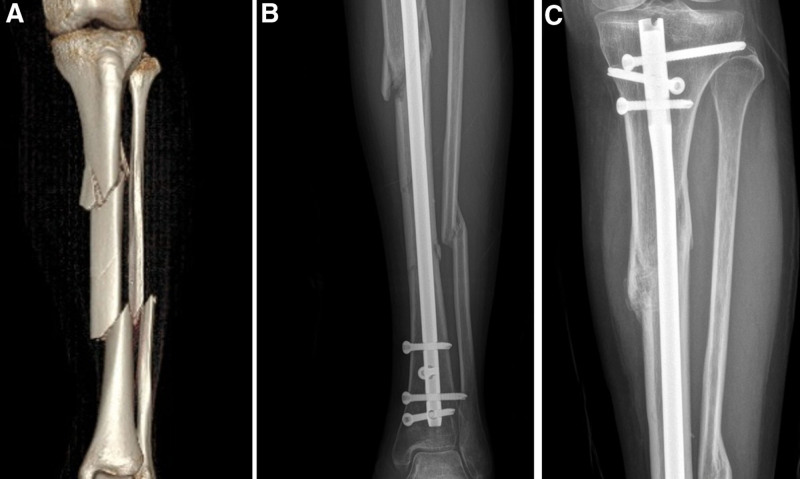
(A) A 49-year-old female patient involved in a traffic accident with STF. (B) Six months after internal fixation with the IMN, X-ray revealed nonunion. (c) Three months after iliac crest bone grafting, X-ray revealed union. IMN = intramedullary nail, STF = segmental tibia fractures.

**Figure 6.**
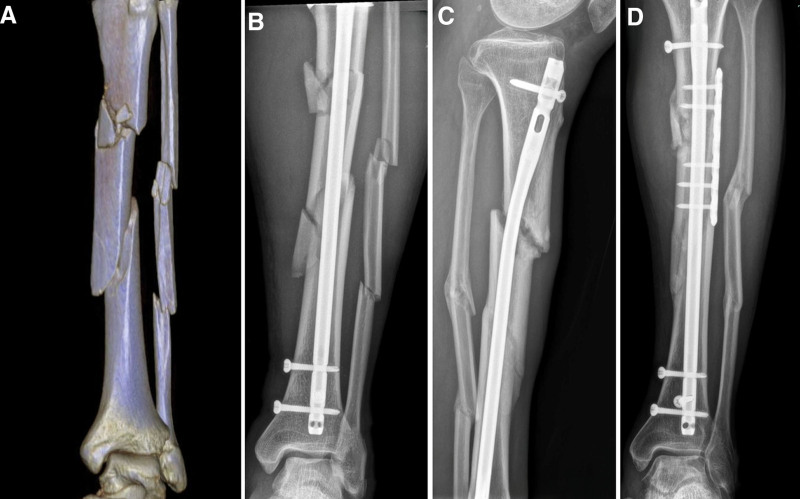
(A) A 63-year-old male patient sustained a left STF. (B) After internal fixation with the IMN, X-ray revealed good axial alignment. (C) Eight months after internal fixation with the IMN, X-ray revealed nonunion occurred in proximal fracture. (D) Two months after iliac crest bone grafting combined with small plate augment fixation, X-ray showed callus formation. IMN = intramedullary nail, STF = segmental tibia fractures.

The characteristics of patients are shown in Table 1. There were no significant differences in sex or age between the 2 groups. As shown in Table 2, although the incision length was longer in the combined group (*P* < .05), the operation time was shorter and the number of fluoroscopies was lower (*P* < .05). There was no significant difference in intraoperative blood loss between the 2 groups. As shown in Table 3, the rate of malunion in the nail group (4, 17.4%) was significantly greater than that in the combination group (0, 0%; Figs. 2 and 3). Meanwhile, time to union was 13.68 ± 4.21 weeks in the combination group which was shorter than that in the nail group (17.56 ± 5.36; *P* < .05; Fig. 4). There was no significant difference in walking ability between the 2 groups. In the nail group, 2 patients with nonunion underwent revision surgery (Figs. 5 and 6). Wound complications occurred in 3 patients, including 2 in the combination group and 1 in the nail group, all of whom healed after wound care. Infection and deep venous thrombosis were not detected.

**Table 1:** Characteristics of the patients.

Variables	Combination group	Nail group	*P* value
Number of patients	18	23	
Gender			
Female	11	15	.79
Male	7	8	
Age (yr)	37.68 ± 25.84	35.42 ± 20.78	.76

**Table 2:** Perioperative parameters of the patients.

Variables	Combination group	Nail group	*P* value
Operation time (min)	96.72 ± 17.21	121.63 ± 20.36	<.05
Incisions length (cm)	26.48 ± 2.01	7.68 ± 1.96	<.05
Fluoroscopy times	14.52 ± 3.21	24.46 ± 5.23	<.05
Intraoperative blood loss (mL)	180.75 ± 50.62	158.85 ± 60.35	.22

**Table 3:** Post-operative results and post-operative complications of 2 groups.

Variables	Combination group	Nail group	*P* value
Time to union (wk)	13.68 ± 4.21	17.56 ± 5.36	<.05
Nonunion	0 (0%)	2 (8.7%)	
Malunion	0 (0%)	4 (17.4%)	
Walking ability	9.1 ± 0.62	8.6 ± 1.35	.15
Wound complication	2 (11.1%)	1 (4.3%)	
Infection	0 (0%)	0 (0%)	
Deep venous thrombosis	0 (0%)	0 (0%)	

## 4. Discussion

Even for experienced orthopedic surgeons, STF are still worth discussing.^[10]^ Owing to the high incidence of malunion and nonunion of STF, satisfactory reduction should be obtained before fixation.^[11]^ Closed or open reduction and internal fixation with IMN is a common method for treating tibial fractures, and reamed IMN offers better bending and rotational stability.^[12]^ In the treatment of STF, the deformity is difficult to completely correct, and temporary reductions are difficult to maintain, resulting in longer operation times and higher rates of malunion. In addition, it is difficult to achieve stable fixation with an IMN alone. Open reduction allows the fracture to be reduced under direct vision, which helps to achieve a faster and more accurate reduction. Therefore, careful open reduction may be a better choice for STF. However, excessive soft tissue dissection should be avoided during the procedure.

The combination of a plate and an IMN is usually used to treat nonunion because it can increase osteosynthesis stiffness, decrease interfragmentary motions, reduce the load on the IMN and promote fracture healing.^[13]^ Previous studies have reported that the combination of a plate and an IMN can be used to treat proximal and distal fractures of the femur and proximal and distal fractures of the tibia.^[14–19]^ However, whether this combination can be used to treat fresh STF remains to be evaluated.

In this study, a total of 41 patients with STF were included, of whom 18 were treated with an IMN assisted by a plate and 23 were treated with an IMN only. In the combination group, there were longer surgical incisions, but the operation time was significantly shorter, and there was no significant difference in intraoperative blood loss between the 2 groups. Despite the large incision, intraoperative bleeding did not increase significantly because of the shortened operation time. Moreover, adequate exposure is beneficial for shortening the operation time. In the combination group, the number of fluoroscopies performed was lower, and the rate of malunion was lower, which was attributed to satisfactory fracture reduction under direct vision and effective maintenance of reduction by the plate. In the combination group, the time to union was shorter and the rate of nonunion was lower, which was attributed to the better stability provided in the combination group, thereby decreasing interfragmentary motions. There are 3 benefits to using plates. First, as a reduction tool, it can effectively prevent the displacement of fractures caused by IMN implantation. Second, it acts as an internal fixation device to reduce the range of motion of intermediate segmental fragments, which is very important in the case of STF. The third is to share part of the stress during postoperative functional exercise to reduce the risk of IMN breakage. Arastu et al reported the risk of rotation displacement of intermediate fragments in STF during reaming, IMN placement, and post-operative functional exercise. They also reported that rotational displacement disrupted the blood supply to intermediate fragments and affected the bone healing process.^[20]^ At the short-term follow-up, there was no statistically significant difference in walking ability between the 2 groups. Moreover, the 2 procedures have similar effects on limb function. However, if these patients are followed for another 10 or 20 years, there may be a different outcome. Due to the 4 malunion patients in the nail group and changes in the mechanical axis of the lower limb, osteoarthritis of the adjacent joint may be complicated in the future, thus affecting the function of the affected limb.

The incidence of malunion and the time to union in the combined group in this study were significantly lower than those reported in the literature. Sohn et al^[21]^ reported that there were 5 cases (13.9%) of axial malalignment in the treatment of STF with IMN, and the time to union was 23.8 ± 14.3 weeks; 4 cases (12.1%) of rotational malalignment in the treatment of STF with plates, and the time to union was 23.9 ± 11.4 weeks. Gamal and Shams^[22]^ reported that the mean time to union for plate treatment of STF was 20 ± 2.22 weeks. Wu and Shih^[23]^ reported that the mean time to union for IMN treatment of STF was 4.5 ± 1.6 months.

Another concern is that open reduction and the insertion of small plates can result in more soft tissue dissection and may increase the incidence of wound complications. Therefore, the incision where the small plate is placed should avoid the damaged skin, and the whole procedure should be performed gently to avoid further aggravation of soft tissue injury. In this study, wound complications occurred in 2 patients in the combination group and 1 patient in the nail group, but all of them healed after wound care. Moreover, our research suggested that plate assisted IMN did not cause more infections or other complications.

Our study has several limitations. First, selection bias is an inevitable inherent shortcoming in retrospective analysis. Second, the single-center nature of the study and the small sample size may have affected the statistical power. A multicenter prospective study is recommended to validate the findings in a larger sample size.

## 5. Conclusion

In summary, our results suggest that locking plates and monocortical locking screws combined with IMN are safe and effective in the treatment of STF. In the future, biomechanical studies will be necessary to verify whether this approach can carry out early weight-bearing.

## Author contributions

**Conceptualization:** Wangsheng Wu, Bingsheng Liu.

**Data curation:** Wangsheng Wu, Huajuan Wang, Qunyang Zheng, Yi Mao, Bingsheng Liu.

**Formal analysis:** Wangsheng Wu, Bingsheng Liu.

**Investigation:** Wangsheng Wu, Huajuan Wang, Qunyang Zheng, Yi Mao.

**Methodology:** Bingsheng Liu.

**Project administration:** Bingsheng Liu.

**Resources:** Wangsheng Wu, Huajuan Wang.

**Software:** Wangsheng Wu, Qunyang Zheng, Yi Mao.

**Validation:** Qunyang Zheng.

**Writing – original draft:** Wangsheng Wu, Huajuan Wang.

**Writing – review & editing:** Bingsheng Liu.

## References

[1] Court-Brown CM Caesar B. Epidemiology of adult fractures: a review. Injury. 2006;37:691–7. 16814787 10.1016/j.injury.2006.04.130

[2] Revak T Mahle P Nicolaou D Watson JT. Permanent reduction plate and intramedullary nailing of open tibia fractures: do we need to take them out. Injury. 2021;52:2439–43. 33879336 10.1016/j.injury.2021.03.056

[3] Coelho FA Sagoo KS Oluku J Cheema KS. Tibial malrotation following intramedullary nailing: a literature review. Cureus. 2021;13:e19683. 34804759 10.7759/cureus.19683 PMC8600094

[4] Kahn KM Beals RK. Malrotation after locked intramedullary tibial nailing: three case reports and review of the literature. J Trauma. 2002;53:549–52. 12352495 10.1097/00005373-200209000-00025

[5] Puloski S Romano C Buckley R Powell J. ) Rotational malalignment of the tibia following reamed intramedullary nail fixation. J Orthop Trauma. 2004;18:397–402. 15289683 10.1097/00005131-200408000-00001

[6] Theriault B Turgeon AF Pelet S. Functional impact of tibial malrotation following intramedullary nailing of tibial shaft fractures. J Bone Joint Surg Am. 2012;94:2033–9. 23172320 10.2106/JBJS.K.00859

[7] Vallier HA Le TT Bedi A. Radiographic and clinical comparisons of distal tibia shaft fractures (4 to 11 cm proximal to the plafond): plating versus intramedullary nailing. J Orthop Trauma. 2008;22:307–11. 18448983 10.1097/BOT.0b013e31816ed974

[8] Goh EL Chidambaram S Eigenmann D Ma S Jones GG. Minimally invasive percutaneous plate osteosynthesis versus intramedullary nail fixation for closed distal tibial fractures: a meta-analysis of the clinical outcomes. SICOT J. 2018;4:58–P.58. 30560779 10.1051/sicotj/2018055 PMC6298240

[9] de Abreu EL Sena CB Saldanha RFS. Effectiveness of treatment of transtrochanteric fractures with dynamic hip screws using minimally invasive access. Rev Bras Ortop. 2016;51:138–42. 27069880 10.1016/j.rboe.2016.01.001 PMC4811999

[10] Teraa M Blokhuis TJ Tang L Leenen LP. Segmental tibial fractures: an infrequent but demanding injury. Clin Orthop Relat Res. 2013;471:2790–6. 23229434 10.1007/s11999-012-2739-z PMC3734401

[11] McMillan TE Johnstone AJ. Technical considerations to avoid delayed and non-union. Injury. 2017;48:S64–8. 28499466 10.1016/j.injury.2017.04.019

[12] Stein H Horesh Z Lerner A. Current trends for the biological treatment of segmental bone loss in high-energy long bone fractures. Orthopedics. 2006;29:773–7. 17004585 10.3928/01477447-20060901-07

[13] Ye J Zheng Q. Augmentative locking compression plate fixation for the management of long bone nonunion after intramedullary nailing. Arch Orthop Trauma Surg. 2012;132:937–40. 22395822 10.1007/s00402-012-1497-4

[14] Galante C Djemetio M Fratus A. Management of distal femoral fractures with metaphyseal and articular comminution (AO/OTA 33C) using nail and plate fixation: a technical note and case series of 14 patients. Eur J Orthop Surg Traumatol. 2023;33:3519–29. 37204623 10.1007/s00590-023-03577-z

[15] Kubiak EN Camuso MR Barei DP Nork SE. Operative treatment of ipsilateral noncontiguous unicondylar tibial plateau and shaft fractures: combining plates and nails. J Orthop Trauma. 2008;22:560–5. 18758288 10.1097/BOT.0b013e318185fa7e

[16] Yoon RS Liporace FA. Intramedullary nail and plate combination fixation for complex distal tibia fractures: when and how. J Orthop Trauma. 2016;30:S17–21. 10.1097/BOT.0000000000000698 27768628

[17] Yoon RS Bible J Marcus MS. Outcomes following combined intramedullary nail and plate fixation for complex tibia fractures: a multi-centre study. Injury. 2015;46:1097–101. 25843886 10.1016/j.injury.2015.03.019

[18] Yoon RS Gage MJ Donegan DJ Liporace FA. Intramedullary nailing and adjunct permanent plate fixation in complex tibia fractures. J Orthop Trauma. 2015;29:e277–9. 25932525 10.1097/BOT.0000000000000299

[19] Xu K Wang G Lu L. Intramedullary nail fixation assisted by locking plate for complex subtrochanteric femur fractures: a retrospective study. J Orthop Sci. 2023;28:1105–12. 35864029 10.1016/j.jos.2022.06.015

[20] Arastu MH Sheehan B Paolucci EO Buckley RE. Does it really spin? Intra-medullary nailing of segmental tibial fractures--a cadaveric study. Injury. 2015;46:643–8. 25627483 10.1016/j.injury.2015.01.014

[21] Sohn HS Chung JY Song HK. Analysis of complications and clinical outcomes in the treatment of segmental tibial fractures according to the method of internal fixation. Asian J Surg. 2018;42:740–5. 30471888 10.1016/j.asjsur.2018.11.001

[22] Gamal O Shams A. Surgical technique for biological fixation of closed segmental tibial fractures by the Less Invasive Stabilization System (LISS). Sicot J. 2018;4:48–P.48. 30427774 10.1051/sicotj/2018046 PMC6424021

[23] Wu CC Shih CH. Segmental tibial shaft fractures treated with interlocking nailing. J Orthop Trauma. 1993;7:468–72. 8229384 10.1097/00005131-199310000-00010

